# Supplementary material for: Pen-drawn Marangoni swimmer
Source: Nat Commun. 2023 Jun 16;14:3597. doi: 10.1038/s41467-023-39186-x (PMC10276010; doi:10.1038/s41467-023-39186-x)
Supplement: Supplementary file 1 — Supplementary Information [file 41467_2023_39186_MOESM1_ESM.pdf]

nature COMMUNICATIONS

Supplementary Information for

Pen-drawn Marangoni swimmer

Seo Woo Song*†, Sumin Lee†, Jun Kyu Choe, Amos Chungwon Lee, Kyoungseob Shin, Junwon Kang, Gyeongjun Kim, Huiran Yeom, Yeongjae Choi, Sunghoon Kwon*, Jiyun Kim*

†These authors contributed equally to this work.
*Correspondence to: seowoo313@snu.ac.kr, skwon@snu.ac.kr, jiyunkim@unist.ac.kr

The PDF file includes:

Supplementary Text
Supplementary Table 1
Supplementary Figs. 1 to 7
References for Supplementary Information

Other Supplementary Information for this manuscript include the following:

Supplementary Movies 1 to 9 (.mp4)
Supplementary Movie Legends

Supplementary Text

Mathematical modeling and FEA simulation for predicting camphor-driven swimmer trajectories

Supplementary Table 1. Parameters for mathematical modeling and FEA simulation

Notation	Description	Unit
$\Gamma(x, y, t)$, $\Gamma(x, t)$, Γ	Surface camphor concentration<br>Notation depends on modeling dimensions	[mol m^{-2}]
$f\ (x, y)$	Supply of the camphor molecules from the camphor engine to the water surface<br>(in 2-dimension modeling)	[mol s^{-1}m^{-1}]
γ	Surface tension	[N m^{-1}]
γ_0	Surface tension of pure water	[N m^{-1}]
p	Positive constant	
$F_{Driving\ force}$	Driving force of the moving camphor object<br>(in 2-dimensional modeling)	[N]
F_{drag}	Drag force exerted on the object<br>(in 2-dimensional modeling)	[N]
D	Diffusion constant	[m^2 s^{-1}]
k	Sum of *k*s and *k*d	[s^{-1}]
ks	Sublimation rate from the water surface	[s^{-1}]

$k\mathrm{d}$	Dissolution rate from the water surface	[s^{-1}]
x_0	Position at the edge of the camphor engine	[m]
m	Mass of the camphor boat	[kg]
μ	Friction coefficient	[N m^{-1} s]
F_w	Driving force of the moving camphor boat<br>(in 1-dimensional modeling)	[N]
F_{w0}	Driving force of the stationary camphor boat<br>(in 1-dimensional modeling)	[N]
L	Length of the contact line between the camphor disk and water surface	[m]
c	Camphor concentration in the bulk	[mol m^{-3}]
R	Gas concstant (=8.31)	[J mol^{-1} K^{-1}]
T	Absolute temperature (=297)	[K]
v_{steady}	Steady state velocity of camphor engine	[m s^{-1}]
α	Supply rate of camphor molecules from the camphor engine to the water surface<br>(in 1-dimensional modeling)	[mol s^{-1}]

Predicting an object's trajectory can be achieved through a comprehensive analysis of the net force and net torque applied to the object. By understanding these factors, it becomes possible to anticipate the motion and behavior of the Marangoni swimmer in various situations.

For example, let's consider a rectangular Marangoni swimmer with two camphor engines placed on one edge, as shown in Supplementary Fig. 1.

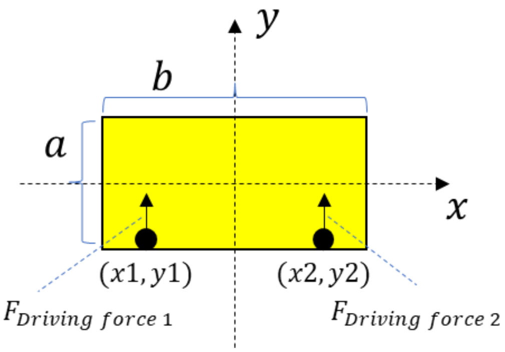

Supplementary Fig. 1. Example of rectangular Marangoni swimmer with two camphor engines placed on one edge.

We refer to the two-dimensional modeling of a Marangoni swimmer's motion studied by H. Kitahata and colleagues [1]. The surface concentration of camphor molecules is defined as $\Gamma(x, y, t)$, which is represented as Γ for simple notation in equation (1). To figure out the trajectories of the swimmer derived by the camphor molecules in two-dimensional space, we need to consider three equations: reaction-diffusion equation for camphor molecules, net force equation, and net torque equation exerted on the swimmer.

$$\frac{\partial \Gamma}{\partial t} = D\nabla^2\Gamma - k\Gamma + f(x, y) \quad (1)$$
$$(\gamma = \gamma_0 - p\Gamma)$$

The first term on the right-hand side depicted surface diffusion of camphor molecules. The second term means the sublimation (from the water surface to the air) and dissolution (from water surface to the bulk water phase) of the camphor molecules. The last term is the supply of the camphor molecules from the camphor engine, where $f(x, y)$ is described as f_0 in the region in two-dimensional space that corresponds to the shape of the camphor engine, and 0 in the other region. We can presume a linear relation between γ and Γ for simplicity. (γ is a surface tension.)

$$\sum F = F_{Driving\ force\ 1} + F_{Driving\ force\ 2} + F_{drag} \quad (2)$$
$$\sum \tau = \tau_{F_{Driving\ force\ 1} + F_{Driving\ force\ 2}} + \tau_{drag} \quad (3)$$

Above equations (2) and (3) are the net force equation and net torque equation. We need to consider the force and torque exerted by the camphor molecules and also include drag forces and drag torques. Then we are able to calculate the trajectory of the Marangoni swimmer by solving the above equations.

Previous literature has extensively discussed the modeling of driving forces generated by camphor engines in various systems [1–7]. When neglecting factors contributing to the system's complexity, such as the interaction between camphor engines, the swimmer's shape, and external forces, the driving force of the camphor engine can be modeled in a linear term in one-dimensional system,

with a direction vector perpendicular to the edge on which the camphor engines are placed. These assumptions neglect the nonlinear terms, fluid dynamics complexities and consider only the dominant forces. As a result, the simplified linear model may provide an approximate prediction. However, these assumptions can simplify the model and reduce the computational complexity of the prediction, making it a useful tool for analyzing the motion of Marangoni swimmers with camphor engines.

The driving force generated by the camphor engine, as shown in Supplementary Fig. 2, can be derived from Reaction-diffusion and Newtonian equations (simplified version in one-dimensional space). Based on the previous report (N. J. Suematsu et al., Langmuir 2014) [8], when solving these two equations, the steady-state swimmers' driving force and velocity have a relationship as expressed in Equation (8). In brief, the driving force F_w is obtained by multiplying the width of the swimmer (L) by the surface pressure ($\gamma_0 - \gamma$). If we solve the reaction-diffusion equation (4) with Gibbs adsorption isotherm (7), and under the assumption that the driving force is balanced by the friction force ($F_w = \mu v_{steady}$), the steady-state driving force for the swimmers can be determined as shown in equation (8). A more detailed solving process can be found in the Suematsu's research [8].

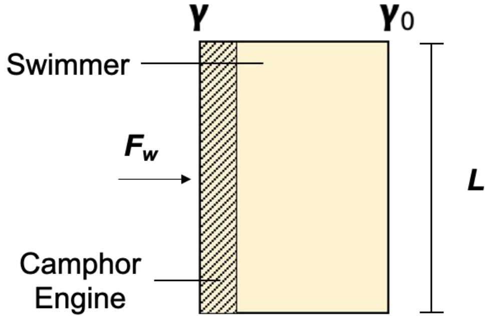

Supplementary Fig. 2. Force diagram on a Marangoni swimmer with a long camphor engine placed on one edge.

Reaction–diffusion equation:

$$\frac{\partial \Gamma(x,t)}{\partial t} = D\nabla^2\Gamma(x,t) - \kappa\Gamma(x,t) + \alpha L^{-1}\delta(x - x_0) \quad (4)$$

where the delta function, δ, denotes the assumption that the camphor molecules are supplied only to the stern of the boat [9].

Newtonian equation:

$$m\frac{d^2x_0}{dt^2} = -\mu\frac{dx_0}{dt} + F_w = 0 \quad (5)$$

$$F_w = L(\gamma_0 - \gamma(\Gamma(x_0,t))) \quad (6)$$

Gibbs adsorption isotherm:

$$\Gamma = -\frac{c}{RT}\left(\frac{\partial\gamma}{\partial c}\right) \quad (7)$$

Driving force at steady state:

$$F_{w0} = \mu v_{steady}\sqrt{\frac{v_{steady}^2}{4kD} + 1} \quad (8)$$

By inputting the calculated driving force into the system, it becomes relatively easy to estimate the object's trajectory. However, due to the difficulties in calculating this approach for every system with varying shapes and designs, we utilized FEA simulation to predict the trajectories of various swimmers.

Commercially available finite element analysis (FEA) software, ABAQUS, was used to predict the trajectories of the swimmers. To derive the driving pressure (driving force per unit area of a camphor engine), we first estimated the approximate value of the friction coefficient (μ) based on the previous research conducted by N. Suematsu and colleagues [8]. We designed an experimental condition that mimics the situation where the drag force becomes dominant (driving force negligible) by using swimmers with not-fully-dried camphor engines, which results in a burst release followed by a weakened release of camphor molecules. The experimental data shows relaxation-like motion after the end of the burst release of camphor molecules from the not-fully-dried camphor engines. At the relaxation interval, the drag force becomes dominant, making the velocity of the Marangoni swimmer decrease exponentially. Then the equation (5) can be reasonably approximated and solved as equation (8), where *m* is the mass of the boat (0.07 g) and v_{init} is the initial velocity of the swimmer.

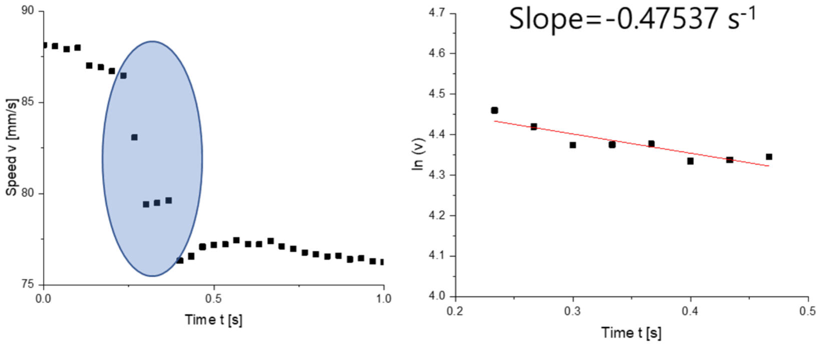

Supplementary Fig. 3. Relaxation-like motion of a swimmer with not-fully-dried camphor engines.

$$v(t) = v_{init}\, e^{-\frac{\mu}{m}t} \quad (8)$$

Then the value of μ (3.327 10^{-5} $\mathrm{Nm^{-1}s}$) can be derived from the slope of $ln(v)$. Subsequently, we calculated the driving force F_{w0} of each camphor engine of different concentrations from their steady-state velocity v_{steady} using the following equation (7).

Because the constants k and D are broadly applicable to a variety of situations regardless of the shape of the swimmer, we used the values k = 2 10^{-2} s^{-1} and D = 4 10^{-3} m^2s^{-1} adopted from a previous report *(N. J. Suematsu et al., Langmuir* 2014) [8]. The driving pressure was then calculated for each concentration by dividing the driving force by the working area (the side area of the swimmer where the camphor ink was drawn, A = 3 mm^2). For the camphor inks applied in dots, we analyzed the diameter of the dots (~ 2.5 mm) and applied the driving pressure to the nearest side of the swimmer with equal length. For the dots located in the vertex, the driving pressure was applied to both nearest sides of the swimmer.
Finally, stagnation pressure P_s was applied to the edges of the swimmer, as computed by the following equation:

$$P_s = -c_s \left(v \cdot n - v_{ref} \cdot n\right)^2 \quad (9)$$

where c_s is the fitted coefficient (c_s = 1.4 kg/m^3) from the circular trajectory in Fig. 2e, n is the normal unit outward from the element where the surface pressure is applied, and v_{ref} is the velocity of the reference node.

In all simulations, the swimmers with length = 20 mm, width = 10 mm, and height = 0.3 mm were constructed with 3D deformable solid elements of type C3D8 using a linear elastic model. The mechanical properties were determined by using the following input parameters: density d = 1.45 g/cm^3, Young's modulus E = 3.275 GPa, and Poisson's ratio v = 0.4.

Supplementary Figures

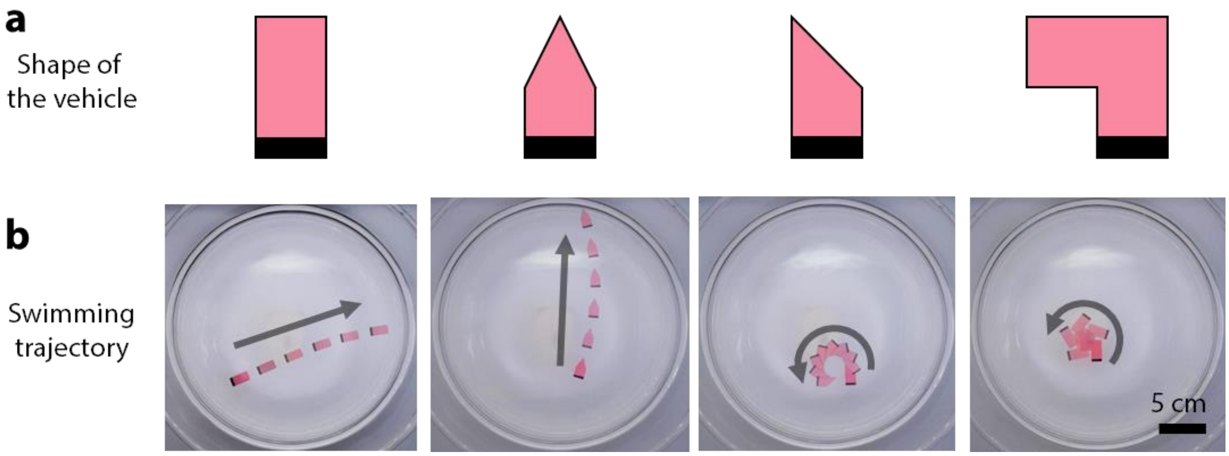

Supplementary Fig. 4. Swimming trajectories according to the shape of the vehicle. a Marangoni swimmers with different shapes of the vehicle. The swimmers in this experiment have vehicles of different shapes but the same pattern of the camphor engine. **b** Trajectories of the swimmers with vehicles of different shapes.

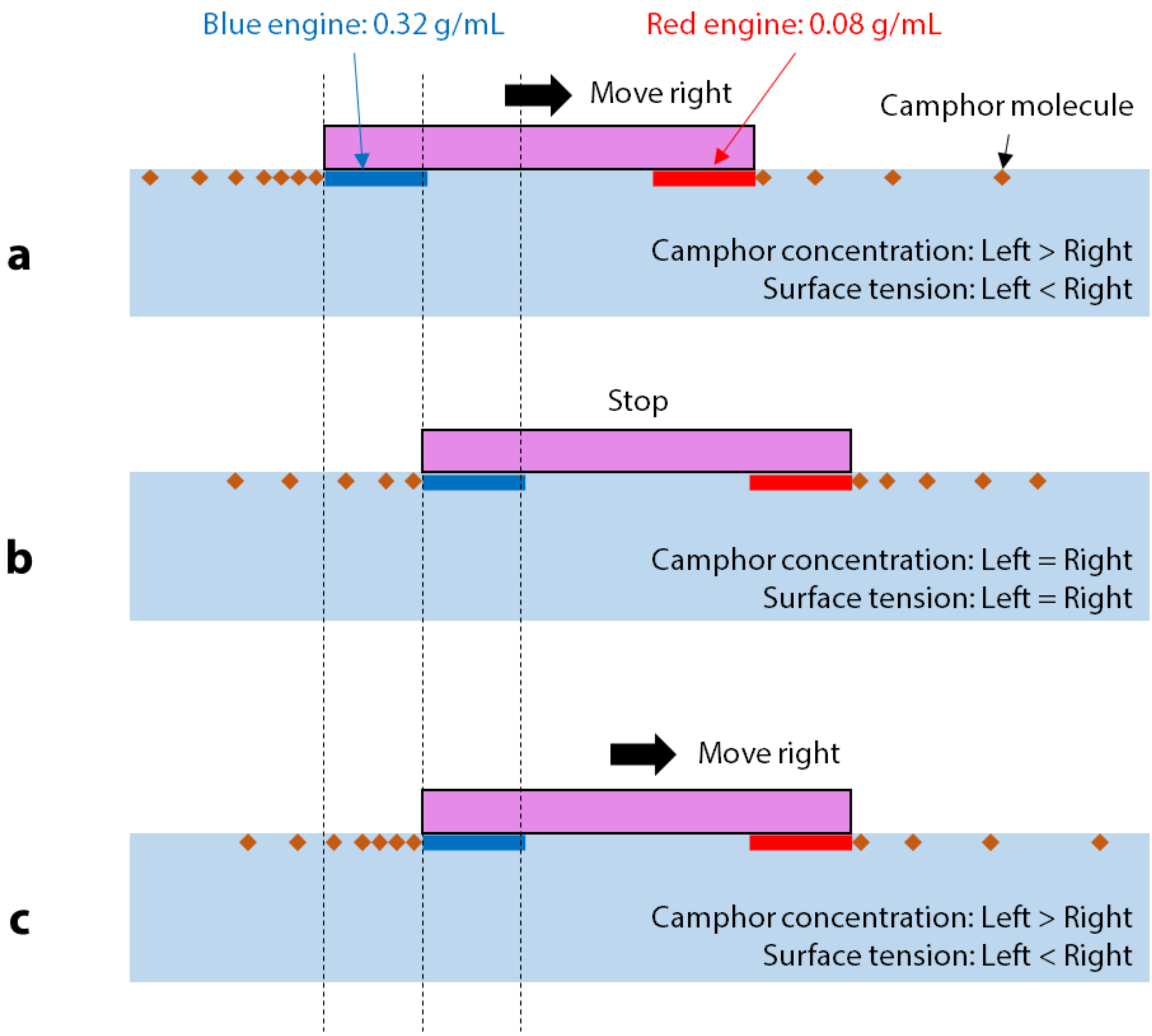

Supplementary Fig. 5. Theoretical model of intermittent movement of an asymmetric Marangoni swimmer. a A blue camphor engine drawn with higher camphor concentration releases the camphor molecules more rapidly. Because camphor concentration on the left side is higher than that on the right side, the swimmer moves toward the right. **b** The camphor molecules on the right side are compressed and those on the left side are dispersed as the swimmer moves to the right. Hence, the camphor concentrations on both sides become equal and the swimmer stops. **c** As the swimmer releases the camphor in place, an imbalance between the camphor concentrations on both sides occurs again and the swimmer moves.

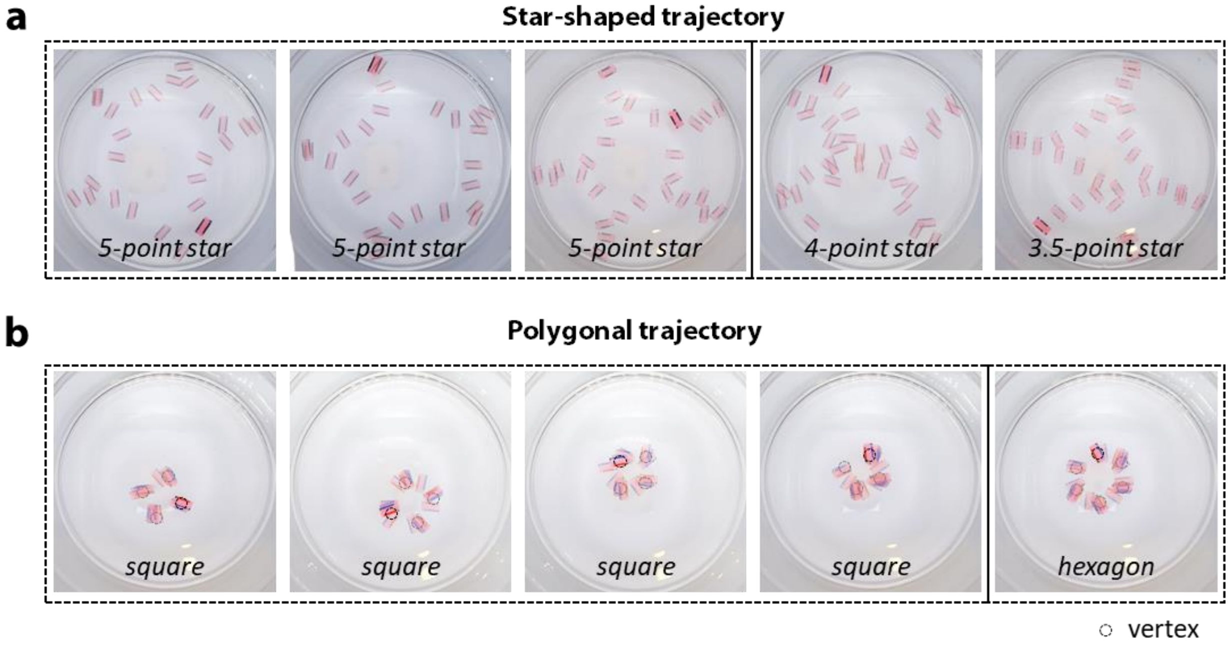

Supplementary Fig. 6. Reproducibility of complex motion programming. a Reproducibility of star-shaped trajectory programming. Three out of five attempts had about five vertices during one round of the boundary of the water tank. The other two trials showed about four vertices per one round. **b** Reproducibility of polygonal trajectory programming. Four out of five attempts showed square like trajectories while the other one trial showed hexagonal trajectory.

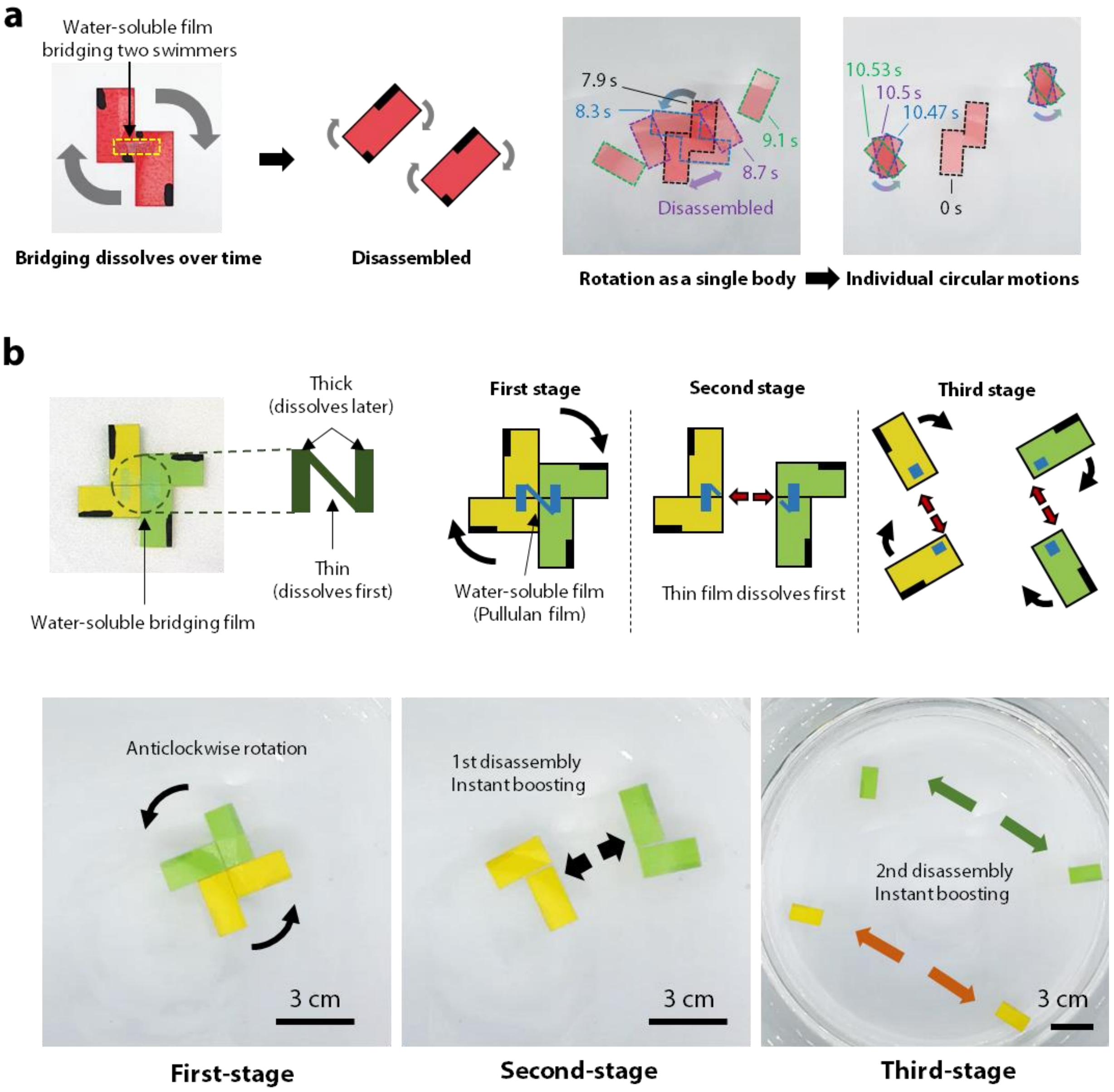

Supplementary Fig. 7. Multistep motion programming using water-soluble bridges. a Two-step motion programming. **b** Three-step motion programming.

References for Supplementary Information

1. Kitahata, H., Iida, K. & Nagayama, M. Spontaneous motion of an elliptic camphor particle. *Phys. Rev. E* **87**, 010901 (2013).
2. Nagayama, M., Nakata, S., Doi, Y. & Hayashima, Y. A theoretical and experimental study on the unidirectional motion of a camphor disk. *Physica D: Nonlinear Phenomena* **194**, 151–165 (2004).
3. Nakata, S., Nagayama, M., Kitahata, H., J. Suematsu, N. & Hasegawa, T. Physicochemical design and analysis of self-propelled objects that are characteristically sensitive to environments. *Physical Chemistry Chemical Physics* **17**, 10326–10338 (2015).
4. Nakata, S. *et al.* Self-Rotation of a Camphor Scraping on Water: New Insight into the Old Problem. *Langmuir* **13**, 4454–4458 (1997).
5. Morohashi, H., Imai, M. & Toyota, T. Construction of a chemical motor-movable frame assembly based on camphor grains using water-floating 3D-printed models. *Chemical Physics Letters* **721**, 104–110 (2019).
6. Kitahata, H., Koyano, Y., J.G. Löffler, R. & Górecki, J. Complexity and bifurcations in the motion of a self-propelled rectangle confined in a circular water chamber. *Physical Chemistry Chemical Physics* **24**, 20326–20335 (2022).
7. Tanaka, S., Sogabe, Y. & Nakata, S. Spontaneous change in trajectory patterns of a self-propelled oil droplet at the air-surfactant solution interface. *Phys. Rev. E* **91**, 032406 (2015).
8. Suematsu, N. J., Sasaki, T., Nakata, S. & Kitahata, H. Quantitative Estimation of the Parameters for Self-Motion Driven by Difference in Surface Tension. *Langmuir* **30**, 8101–8108 (2014).

9. Suematsu, N. J., Nakata, S., Awazu, A. & Nishimori, H. Collective behavior of inanimate boats. *Phys. Rev. E* **81**, 056210 (2010).
