# Supplementary material for: Pen-drawn Marangoni swimmer
Source: Nat Commun. 2023 Jun 16;14:3597. doi: 10.1038/s41467-023-39186-x (PMC10276010; doi:10.1038/s41467-023-39186-x)
Supplement: Supplementary file 3 — Description of Additional Supplementary Files [file 41467_2023_39186_MOESM3_ESM.docx]

**File Name: Supplementary Movie 1.**Description: Overview of pen-drawn Marangoni swimmers. This video is provided with audio explanations.

**File Name: Supplementary Movie 2.**Description: Quantitative analysis of swimmers’ speed using ImageJ. ‘Mosaic’ plugin in ImageJ was used for quantitative analysis of the swimmers’ speed.

**File Name: Supplementary Movie 3.**Description: Controllable motion of pen-drawing Marangoni swimmer. This movie presents the results of the experiments and simulations shown in Fig. 2e and f. The time interval between successive frames in Fig. 2e and f is 0.33 s.

**File Name: Supplementary Movie 4.**Description: Complex motion programming by combining different functional modules. This movie presents the results of the experiments shown in Fig. 3a and b. The programming of a star-shaped trajectory and square trajectory is demonstrated.

**File Name: Supplementary Movie 5.**Description: Practical applications of functional module combination for complex motion programming. This movie presents the results of the experiments shown in Fig. 4. The programming of a pathfinding swimmer and a robot soccer player is demonstrated.

**File Name: Supplementary Movie 6.**Description: Versatile applicability of pen-drawn Marangoni swimmer on various substrates. This movie presents the results of the experiments shown in Fig. 5. The applications of a pen-drawn camphor engine on an acrylic plate, paper origami, leaf, and 3D printed structures are demonstrated.

**File Name: Supplementary Movie 7.**Description: Multiple leaf swimmers. Fabrication of pen-drawn Marangoni swimmers on leaves is demonstrated. Each leaf swimmer rotates clockwise or anticlockwise depending on the camphor engine pattern. The swimmers tend to maintain a distance between themselves owing to the decreased surface tension around each swimmer.

**File Name: Supplementary Movie 8.**Description: Multistep motion programming with paper as a time-dependent transformable substrate. This movie presents the results of the experiments shown in Fig. 6. Three different swimming modes of a rocket-shaped swimmer, depending on the engine arrangement, were demonstrated. As the folded region contacts the water surface over time, the activated second-stage engine changes the motion of the swimmer.

**File Name: Supplementary Movie 9.**Description: Multistep motion programming using water-soluble bridges and its application to cargo delivery. This movie presents the results of the experiments shown in Fig. 7. The water-soluble bridging film enables the disassembly of swimmers in a time-dependent manner in addition to multistep motion programming. A swimmer capable of delivering cargo to the target location and returning to the original location is demonstrated by using this property.
